# Hemoglobin Determination Using Pulse Co-Oximetry and Reduced-Volume Blood Gas Analysis in the Critically Ill: A Prospective Cohort Study

**DOI:** 10.3390/diagnostics12122908

**Published:** 2022-11-22

**Authors:** Piotr F. Czempik, Michał P. Pluta, Łukasz J. Krzych

**Affiliations:** 1 Department of Anaesthesiology and Intensive Care, Faculty of Medical Sciences in Katowice, Medical University of Silesia, 40-055 Katowice, Poland; 2 Transfusion Committee, University Clinical Center of the Medical University of Silesia in Katowice, 40-055 Katowice, Poland

**Keywords:** anemia, blood gas analysis, critically ill, co-oximetry, diagnostics, patient blood management

## Abstract

Hospital-acquired anemia is common in patients hospitalized in the intensive care unit (ICU). A major source of iatrogenic blood loss in the ICU is the withdrawal of blood for laboratory testing. The aim of our study was to analyze the feasibility and accuracy of non-invasive spot-check pulse co-oximetry (SpHb), and a reduced-volume blood gas analysis (ABG Hb) for the determination of Hb concentration in critically ill patients. Comparisons between Hb determined with test devices and the gold standard—complete blood count (CBC)—were performed using Bland–Altman analysis and concordance correlation coefficient (CCC). The limits of agreement between SpHb and CBC Hb were –2.0 [95%CI −2.3–(−1.7)] to 3.6 (95%CI 3.3–3.9) g/dL. The limits of agreement between ABG Hb and CBC Hb were −0.6 [95%CI −0.7–(−0.4)] to 2.0 (95%CI 1.9–2.2) g/dL. Spearman’s coefficient and CCC between ABG Hb and CBC Hb were 0.96 (95%CI 0.95–0.97, *p* < 0.001) and 0.91 (95%CI 0.88–0.92), respectively. Non-invasive spot-check Hb co-oximetry is not sufficiently accurate for the monitoring of hemoglobin concentration in critically ill patients. Reduced volume arterial blood gas analysis has acceptable accuracy and could replace complete blood count for the monitoring of Hb concentration in critically ill patients, leading to a significant reduction in blood volume lost for anemia diagnostics.

## 1. Introduction

Anemia is common in patients hospitalized in the intensive care unit (ICU). The prevalence of anemia in patients at admission to the ICU can reach up to 66% [1], and almost all patients become anemic within the next 72 h [2]. In the prospective cohort study by Thomas et al., the incidence of anemia in the ICU was as high as 98% [3]. Anemia that develops during a patient’s stay in hospital is named hospital-acquired anemia (HAA). It was reported that, 75% of non-anemic patients develop HAA during hospitalization. Hospital-acquired anemia contributes to the prolongation of hospital stays and increased mortality [4]. As virtually all patients being admitted to the ICU are anemic or at risk of anemia, the presence of anemia should always be sought at admission and throughout hospitalization in the ICU. Preventive measures against HAA should be introduced in this vulnerable patient population from day one of ICU hospitalization. One of the significant causes of anemia is iatrogenic blood loss. A major source of iatrogenic blood loss in the ICU is the withdrawal of blood for laboratory testing, which may amount to significant quantities [5]. Even one-third of all RBC transfusions in the ICU are due to phlebotomy [6]. However, the volume of blood lost for laboratory diagnostics in the ICU can be reduced through the use of a closed phlebotomy system [7].

The gold standard for hemoglobin (Hb) determination is a complete blood count (CBC) test. Blood for this test is collected in the test tube with ethylenediaminetetraacetic acid (EDTA). The minimal EDTA test tube volume for adults in our institution is 2 mL. Multiple CBC tests performed throughout ICU hospitalization may accumulate to non-negligible blood volumes. Conservation of patients’ own blood through multiple interventions is a cornerstone of patient blood management (PBM) [8]. Apart from the causal treatment of anemia and harnessing tolerance of anemia, an important intervention here is the minimization of iatrogenic blood loss. Therefore, non-invasive monitoring of Hb concentration is an interesting development with potential advantages. 

Non-invasive measurement of Hb concentration provides immediate and repeated results, carries no risk of infection, and in the context of iatrogenic blood loss, there is no need to remove the patient’s blood. The continuous non-invasive measurement of Hb concentration can be performed with a pulse co-oximeter. This method was tested in different clinical scenarios: emergency room [9], complex spine surgery [10], major abdominal and pelvic surgery [11], liver transplantation [12], trauma [13], neurosurgery [14], cardiopulmonary bypass [15], and pediatrics [16]. To our knowledge, there was only one study performed on ICU patients, comparing continuous pulse co-oximetry to three different invasive methods [17]. The authors of the study showed that continuous non-invasive measurement of Hb had similar accuracy to invasive point-of-care methods such as HemoCue^®^ 301 or a blood gas analyzer. All previous studies tested the accuracy of continuous pulse co-oximetry. There was only one study analyzing the accuracy of spot-check Hb pulse co-oximetry in the outpatient setting, which showed wide limits of agreement (LOA) of 4 g/dL, and therefore limited accuracy [18]. There was one study comparing spot-check Hb pulse co-oximetry in critical care and surgical populations which showed limited accuracy in comparison to the laboratory method (LOA 4 g/dL) [19]. Therefore, the accuracy of spot-check Hb pulse co-oximeter in critically ill patients is still unresolved.

Another important measure to minimize blood loss for anemia diagnostics is the use of point-of-care blood gas analysis. The standard volume of a heparinized test tube in our institution is 1 mL, but the amount of blood sampled by a blood gas analyzer is only 0.2 mL. Using the minimal volume of blood to run the laboratory test is another preventive measure against HAA. To our knowledge, there was no study assessing the accuracy of reduced-volume blood gas analysis for Hb determination.

The aim of our study was to analyze the feasibility and accuracy of non-invasive spot-check pulse co-oximetry and a reduced-volume blood gas analysis for the determination of Hb concentration in critically ill patients in order to reduce iatrogenic blood loss.

## 2. Materials and Methods

### 2.1. PBM Measures in the Local ICU

Our previous analysis of inpatient recipients of red blood cells (RBCs) showed that RBCs were most frequently transfused in patients hospitalized in the ICU, whereas more than half of all RBCs were transfused in patients hospitalized in the ICU and surgical departments [20]. According to the principles of PBM, every effort is made in our local ICU to reduce the amount of blood lost for laboratory diagnostics. Only tests that can potentially have an impact on the management of patients are ordered, and if possible, tests are ordered together and determined from a single test tube. To obtain blood samples through an arterial cannula, or a central venous catheter/pulmonary artery catheter, we use a closed technique in which there is no blood discarded. In this technique, blood is aspirated through a connecting tubing well past a sampling three-way stopcock, then blood is withdrawn through a stopcock by either aspiration for point-of-care blood gas analysis, or by using a vacuum system (BD Vacutainer One Use Holder, Becton, Dickinson, and Company, Franklin Lakes, NJ, USA) and a Luer adapter (Vacuette^®^ Luer Adapter, Becton, Dickinson, and Company, Franklin Lakes, NJ, USA) for central laboratory analysis. Therefore, the amount of blood withdrawn for a blood gas analysis or laboratory testing equals the volume of test tubes used.

### 2.2. Patients

We performed a prospective analysis of all consecutive patients admitted to our ICU from 1 March 2022 to 24 July 2022. The local ICU is a 10-bed mixed medical–surgical unit, located in an academic tertiary care medical center with 644 hospital beds. As the aim of the study was to analyze the feasibility and accuracy of two alternative methods of Hb determination in the general ICU population, we planned that there would be no patient exclusions. Demographic, clinical, and laboratory data of the study subjects were retrieved from the study subjects’ electronic health records (AMMS, Asseco Medical Solutions, Rzeszów, Poland). 

### 2.3. Hb Measurements

Every time a patient was in need of simultaneous determination of CBC and ABG, as judged by an attending physician, Hb concentration was measured using 3 different methods. As a non-invasive device, we used a pulse co-oximeter spot-check device (Radical-67, Masimo Corporation, Discovery Irvine, CA, USA) with the latest software version. Pulse co-oximetry utilizes a multiwavelength (8 waves) sensor which acquires data on blood constituents based on light absorption through a finger probe. Based on light attenuation, the device calculates Hb concentration. Every time the device’s maximal sensitivity mode was used. The sensor of the pulse co-oximeter was placed on the fifth finger according to the manufacturer’s instructions. We never used a standard pulse oximeter and a pulse co-oximeter on the same hand in order to minimize interference. In order to make comparisons between devices even more accurate, the pulse co-oximeter sensor was preferentially placed on the same side where a radial artery cannula was sited, through which blood was drawn for Hb determination. We recorded both non-invasively measured Hb concentration (SpHb) and the quality of finger perfusion (perfusion index—PI). Adequate perfusion in the extremity is essential for the Radical-67 to function properly. According to the manufacturer, the lowest PI at which SpHb determination is possible is 0.3. We recorded instances in which there was a need to place the sensor contra-laterally due to poor reading. We also recorded instances in which the device did not produce a reading and the probable reason for this. After Hb was acquired non-invasively, blood was drawn immediately through a 20-gauge radial artery cannula (BD Arterial Cannula, Becton, Dickinson and Company, Franklin Lakes, NJ, USA) in a 2 mL EDTA (K2EDTA) test tube (BD Vacutainer, Becton Dickinson, Franklin Lakes, NJ, UK), thoroughly mixed, then sent immediately to the central laboratory for analysis by a hematology analyzer (XN-1000, Sysmex, Kobe, Japan). The Sysmex XN-1000 series hematology analyzer measures hemoglobin by colorimetry using the cyanide-free, sodium lauryl sulfate method. Then arterial blood gas (ABG) was drawn in a standard heparinized 1mL collection tube (BD A-Line; Becton, Dickinson, and Company, Plymouth, UK) filled to half of its original volume, i.e., 0.5 mL, then processed by a blood gas analyzer located in the ICU (RAPID Point^®^ 500, Siemens, Erlangen, Federal Republic of Germany) with minimal time delay. For both these tests, we recorded Hb concentration and hematocrit (Hct). As it was reported that changes in oxygenation status may have an impact on the accuracy of non-invasive measurement of Hb [20], we also recorded peripheral oxygen saturation (SpO_2_) measured with Masimo Radical-67, and oxygen saturation determined in an ABG sample (SaO_2_). Because low Hb concentrations may also have an impact the accuracy of non-invasively acquired concentration [11], we verified these observations in a subgroup with Hb ≤ 7 g/dL.

### 2.4. Statistical Analysis

The statistical analysis was performed using licensed statistical software (Medcalc v. 18, Ostend, Belgium). Categorical variables were presented as absolute values and percentages. Continuous variables were presented using medians and interquartile ranges (IQR; 25–75 pc.). Comparisons between Hb determined with test devices were performed using Bland–Altman analysis and concordance correlation coefficient (CCC). We measured the limits of agreement (LOA) between the test devices, as it was shown that LOA is the most reliable indicator of accuracy in the clinical setting [21]. The 95% LOA was calculated as the mean of the two values, minus and plus 1.96 standard deviations. The percentage of error was calculated as the difference between the experimental and theoretical values, divided by the theoretical value, and multiplied by 100 to obtain the percentage. Correlations between Hb determined with test devices were assessed using the Spearman’s coefficient and the coefficient of determination (R2) [22] In sub-analysis, we stratified the comparison data according to Hb concentration, ≤7 g/dL being the most firmly established RBC transfusion trigger in the critically ill [23,24]. The criterion of statistical significance was *p* < 0.05.

### 2.5. Ethics Committee Approval

It is important to mention that our study did not increase the amount of blood lost for laboratory analysis, as both of the invasive tests that we used in our study were deemed necessary by an attending physician for the proper management of patients. Due to the observational nature of the study, the local ethics committee decided that the study does not require ethics committee approval (PCN/CBN/0022/KB/292/21).

## 3. Results

The study population included 111 (44%) women and 143 (56%) men. The study population characteristics are presented in Table 1.

We performed 254 comparisons between the three methods. The position of the sensor had to be changed 18 times (no reading). We could not pick up any pulse co-oximetry signal 20 times due to the presence of an artificial nail (*n* = 3) and peripheral hypoperfusion (*n* = 17).

### 3.1. Comparison between SpHb and CBC Hb

**Figure 1:**
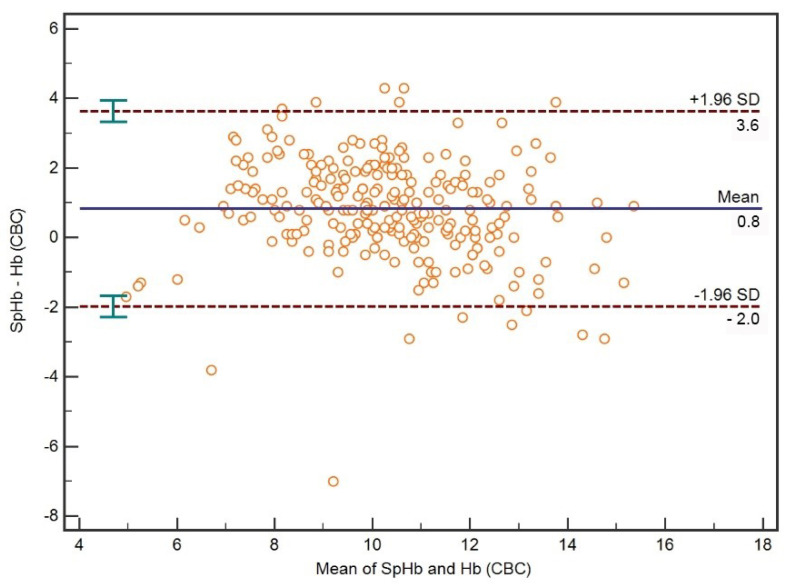
A Bland–Altman plot with mean bias and limits of agreement for all Hb concentrations measured with pulse co-oximetry (SpHb) and hematology analyzer (CBC Hb).

A Bland–Altman plot for all Hb concentrations measured with a pulse co-oximeter (SpHb) and hematology analyzer (CBC Hb) is presented in Figure 1. On average, SpHb was 0.8 (95%CI 0.6–0.9) g/dL (mean bias) higher than CBC Hb. The median absolute percentage error between SpHb and CBC Hb was 11.0 (95%CI 4.8–21.3)%. The limits of agreement between SpHb and CBC Hb were –2.0 [95%CI −2.3–(−1.7)] to 3.6 (95%CI 3.3–3.9) g/dL. 

**Figure 2:**
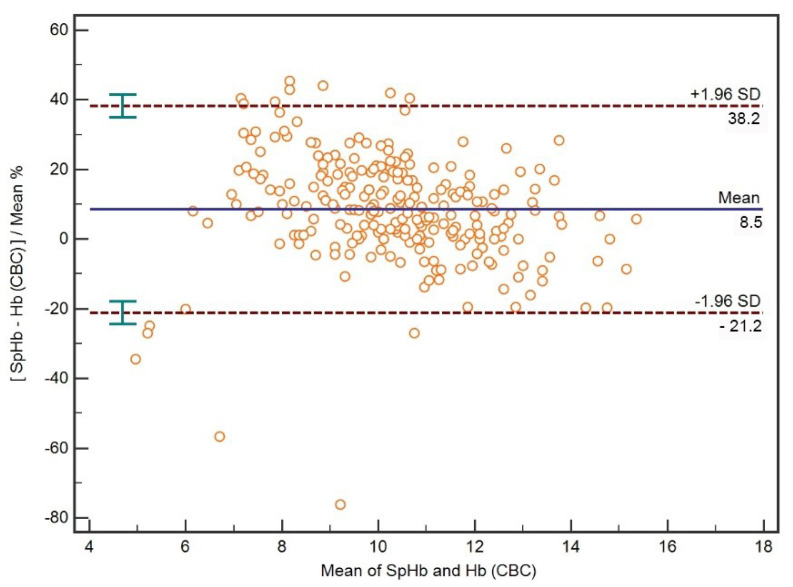
A Bland–Altman plot with mean bias and limits of agreement of percentage differences between Hb concentrations measured with pulse co-oximetry (SpHb) and hematology analyzer (CBC Hb).

A Bland–Altman plot for all percentage differences between mean SpHb and CBC Hb is presented in Figure 2. The limits of agreement between SpHb and CBC Hb expressed as the percentage difference between mean Hb concentrations were −21.2–38.2%.

### 3.2. Comparison between ABG Hb and CBC Hb

**Figure 3:**
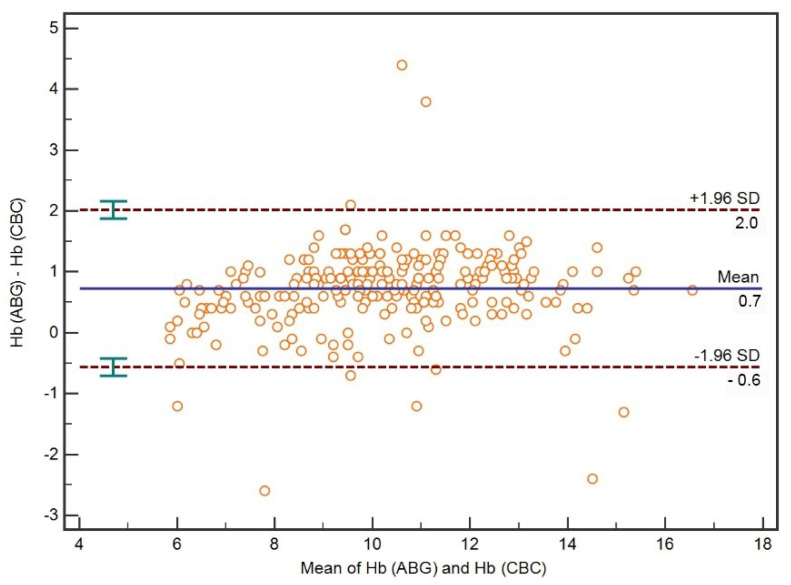
A Bland–Altman plot with mean bias and limits of agreement for all Hb concentrations measured with ABG analysis (ABG Hb) and hematology analyzer (CBC Hb).

A Bland–Altman plot for all ABG Hb and CBC Hb is presented in Figure 3. On average, ABG Hb was 0.7 (95%CI 0.6–0.8) g/dL (mean bias) higher than CBC Hb. The median absolute percentage error between the two methods was 7.1 (95%CI 4.5–9.5)%. The limits of agreement between ABG Hb and CBC Hb were −0.6 [95%CI −0.7–(−0.4)] to 2.0 (95%CI 1.9–2.2) g/dL.

**Figure 4:**
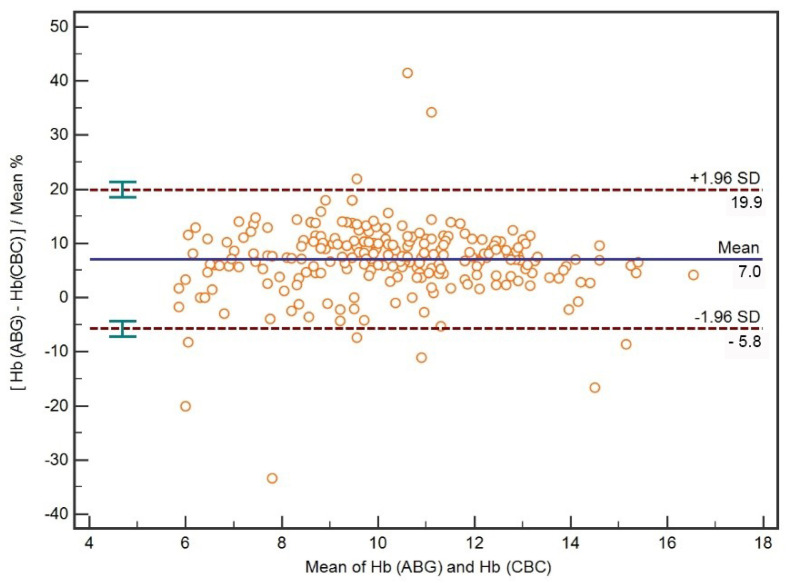
A Bland–Altman plot with mean bias and limits of agreement of percentage differences between Hb concentrations measured with ABG analysis (ABG Hb) and hematology analyzer (CBC Hb).

A Bland–Altman plot for all percentage differences between mean ABG Hb and CBC Hb is presented in Figure 4. The limits of agreement between ABG Hb and CBC Hb expressed as the percentage difference between mean Hb concentrations were −5.8–19.9%.

There were statistically significant correlations between the methods used. All association analyses performed in the study are presented in Table 2.

**Figure 5:**
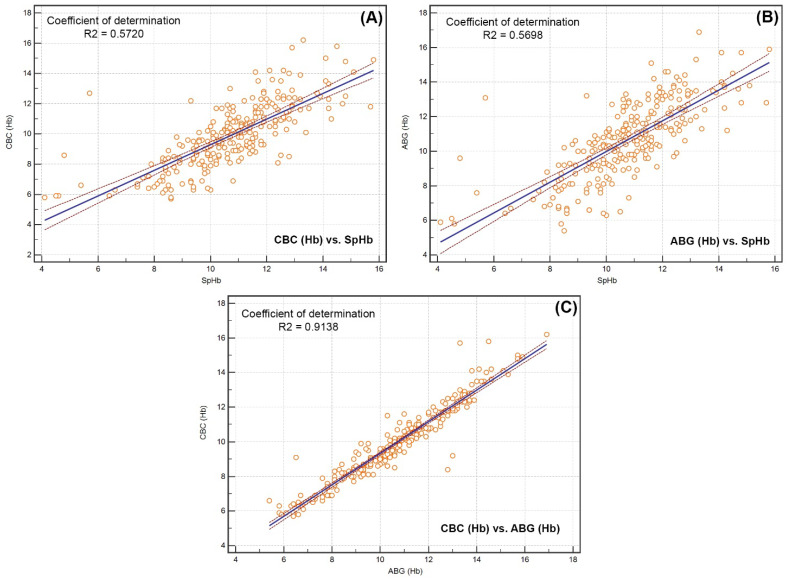
The values of the coefficient of determination between the examined variables: CBC Hb and SpHb (**A**), ABG Hb and SpHb (**B**), CBC Hb and ABG Hb (**C**).

The highest coefficient of determination was observed between the values of CBC-Hb and ABG-Hb (R2 = 0.91) (Figure 5).

### 3.3. Hb Concentration Subgroup Analysis

There were 14 (6%) women and 14 (6%) men in the CBC Hb ≤ 7 g/dL subgroup. The subgroup population characteristics are presented in Table 3.

On average SpHb was 1.6 (95%CI 1.0–2.2) g/dL (mean bias) higher than CBC Hb. The median absolute percentage error between SpHb and CBC Hb was 31.3 (95%CI 21.9–36.7)%. The limits of agreement between SpHb and CBC Hb were −1.4 [95%CI −2.5–(−0.4)] to 4.7 (95%CI 3.6–5.7) g/dL. The limits of agreement between SpHb and CBC Hb expressed as the percentage difference between mean Hb concentrations were −23.8–63.2%.

On average, ABG Hb was by 0.4 (95%CI 0.2–0.6) g/dL (mean bias) higher than CBC Hb. The median absolute percentage error between ABG Hb and CBC Hb was 8.3 (95%CI 4.7–12.0)%. The limits of agreement between ABG Hb and CBC Hb were −0.6 [95%CI −0.9–(−0.3)] to 1.3 (95%CI 1.0–1.7) g/dL. The limits of agreement between ABG Hb and CBC Hb expressed as the percentage difference between mean Hb concentrations were −9.4–20.0%.

## 4. Discussion

The standard CBC test tube has a volume of 2 mL. Multiple CBC tests taken during ICU hospitalization may lead to non-negligible blood volumes. In our study, we tested alternatives to CBC (gold standard) methods of Hb determination in the context of iatrogenic blood loss. Accurate Hb concentration determination is important, as transfusion decisions are partly based on Hb concentration. According to multiple studies, restrictive transfusions (≤7 g/dL) are safe and lead to reduced exposure to RBC [3]. A restrictive transfusion strategy is particularly important in the cohort of critically ill patients in whom potential side effects of transfusion are more deleterious. Therefore, the aim of our study was to assess the feasibility and accuracy of a pulse co-oximetry and reduced-volume blood gas analysis for hemoglobin determination in critically ill patients.

### 4.1. Feasibility of SpHb

As far as the feasibility of pulse co-oximetry is concerned, we were not able to measure Hb 17 out of 254 times (7%) due to the presence of an artificial nail (*n* = 3) and peripheral hypoperfusion (*n* = 18). In the study by Frasca, 3 patients out of 65 had to be excluded due to the inability to obtain a pulse reading (5%) [17]. This result was very close to our data. As opposed to the study by Frasca, we did not record the use and dose of vasopressors in our study; however, we were most interested in how often the non-invasive measurement will be possible in our cohort. Both studies showed that the use of pulse co-oximetry is feasible in critically ill patients; however, both studies used different non-invasive sensors (continuous vs. stop-check). In the ICU study using a spot-check Hb, the measurement of Hb was not possible in 2% of patients due to peripheral artery disease and atrial fibrillation [19].

### 4.2. Accuracy of SpHb

The important question that we wanted to answer in our study was the accuracy of the spot-check Hb pulse co-oximeter. In our study, we showed wide LOA for SpHb and the reference method (−2 to 3.6 g/dL in general). This result was unacceptably high for SpHb as a diagnostic test of Hb concentration determination. The meta-analysis of studies performed in the perioperative setting showed that non-invasive hemoglobin measurement has acceptable accuracy in comparison with lab-based methods [25]. The LOA of ICU patients in the study by Frasca was much narrower (−0.9 to 1 g/dL) than in our study. Although in our study, we used a different sensor (spot-check measurement), we do not know why such a wide LOA was shown in our study. The patients in Frasca et al.’s study were younger; however, their severity of disease was similar to our patients (SAPS II 46 vs. 45). The discrepancy with our study could not be explained by the quality of peripheral perfusion, as these authors also showed good accuracy of SpHb in patients with PI < 0.5 and doses of norepinephrine above 0.2 mcg/kg/min [17]. The quality of peripheral perfusion is not a major factor that has had an impact on the accuracy of results obtained, as long as a pulse reading is produced. In our study, we had to change the site of monitoring 17 times, as we could not obtain a pulse reading. This could be caused by impaired peripheral perfusion to the hand by the presence of an arterial cannula; however, we did not observe any episodes of clinically meaningful distal ischemia in these patients. Nevertheless, pulse co-oximetry reading was present on the contralateral hand and measurement was possible. The SpHb accuracy in our study was worse in the ≤7 g/dL Hb range than in all patients (LOA −1.4 to 4.7 vs. −2.0 to 3.6 g/dL). In the study of ICU patients by DeBarros et al., using a spot-check Hb monitor, there was a significant difference in precision between the Protno-7 monitor and the laboratory hematology analyzer [19]. It was reported that changes in oxygenation status may have an impact on the on accuracy of non-invasive measurement of Hb. The study by Gomma et al. showed that changes in oxygenation in normal volunteers are associated with short-term SpHb signal loss but have no impact on the measured Hb [21]. We did not observe any major oxygenation drops in our patients that could have an impact on the feasibility of the accuracy of SpHb.

### 4.3. Accuracy of ABG Hb

In our study, we showed acceptable accuracy of reduced-volume (0.5 mL) ABG analysis for determination of Hb compared to a hematology analyzer (LOA −0.6 to 2.0 g/dL). However, our results are two times less accurate than in the study by Frasca et al., who showed good accuracy of ABG in the determination of Hb concentration compared to a hematology analyzer (LOA 0.3–1.6 g/dL) [17].

### 4.4. Study Limitations

We performed our study in a single ICU, in a single institution, and that could have had an impact on the results obtained. However, we tried to keep the maximal objectivity of results obtained by following manufacturers’ instructions for all three devices and keeping delay times minimal. Moreover, the SpHb measurements were performed by only two researchers, decreasing interindividual variability.

One limitation of pulse co-oximetry is that it relies on peripheral perfusion. In patients with poor peripheral perfusion, the obtained results are not reliable enough to be clinically useful. We did not record the presence or doses of vasopressors in the study population. As our study was a pragmatic one, analyzing the feasibility and accuracy of non-invasive Hb measurements, we decided not to record all possible factors that could affect the quality of signal picked up by a pulse co-oximeter. Nevertheless, for every non-invasive measurement, we recorded the value of a proprietary perfusion index (PI), which is the numerical index of perfusion adequacy (the final product of perfusion in the finger and factors that have an impact on it). However, from our research and that by Frasca et al., we know that peripheral perfusion has no significant impact on the accuracy of the results [17].

## 5. Conclusions

Non-invasive spot-check Hb co-oximetry is not sufficiently accurate for monitoring of hemoglobin concentration in critically ill patients. Reduced volume arterial blood gas analysis has acceptable accuracy and could replace complete blood count for monitoring of hemoglobin concentration in critically ill patients, leading to a significant reduction in blood volume lost for anemia diagnostics. It could, therefore, be an important measure of Patient Blood Management in the intensive care unit.

## Figures and Tables

**Table 1:** The study population characteristics.

Parameter	Value, Median, IQR ^1^
Age [years]	63 (45–70)
CBC ^2^ Hb ^3^ [g/dL]	9.8 (8.5–11.4)
ABG ^4^ Hb [g/dL]	10.7 (9.2–12.4)
SpHb ^5^ [g/dL]	10.8 (9.7–11.9)
CBC Hct ^6^ [%]	30.6 (26.2–34.5)
ABG Hct [%]	31.0 (27.0–36.0)
SaO_2_ ^7^ [%]	97 (95–98)
SpO_2_ ^8^ [%]	96 (94–98)
PI ^9^	3.1 (1.3–5.7)

^1^ Interquartile range; ^2^ complete blood count; ^3^ hemoglobin; ^4^ arterial blood gas; ^5^ non-invasive hemoglobin determination; ^6^ hematocrit; ^7^ arterial oxygen saturation; ^8^ peripheral oxygen saturation; ^9^ perfusion index.

**Table 2:** Association analyses for the parameters measured in the study population.

Comparisons	Spearman’s Coefficient (95%CI ^1^)	*p*	Concordance Correlation Coefficient (95%CI ^1^)
SpHb ^2^-CBC ^3^ Hb ^4^	0.78 (0.73–0.83)	<0.001	0.69 (0.63–0.75)
SpHb-CBC Hb (Hb ≤ 7 g/dL)	0.34 (−0.04–0.63)	0.1	0.11 (0.02–0.20)
ABG ^5^ Hb-CBC Hb	0.96 (0.95–0.97)	<0.001	0.91 (0.88–0.92)
ABG Hb-CBC Hb (Hb ≤ 7 g/dL)	0.74 (0.51–0.87)	<0.001	0.47 (0.24–0.64)
ABG Hct ^6^-CBC Hct	0.91 (0.89–0.93)	<0.001	0.86 (0.83–0.89)
ABG Hct-CBC Hct (Hb ≤ 7 g/dL)	0.71 (0.45–0.85)	<0.001	0.65 (0.40–0.81)
SpHb-ABG Hb	0.78 (0.73–0.83)	<0.001	0.74 (0.69–0.79)
SpHb-ABG Hb (Hb ≤ 7 g/dL)	0.17 (−0.21–0.51)	0.4	0.15 (−0.02–0.3)

^1^ Confidence interval; ^2^ non-invasive hemoglobin determination; ^3^ complete blood count; ^4^ hemoglobin; ^5^ arterial blood gas; ^6^ hematocrit.

**Table 3:** The Hb ≤ 7 g/dL subgroup population characteristics.

Parameter	Value, Median, IQR ^1^
Age [years]	66 (51–73)
CBC ^2^ Hb ^3^ [g/dL]	6.5 (6.2–6.7)
ABG ^4^ Hb [g/dL]	6.7 (6.4–7.3)
SpHb ^5^ [g/dL]	8.4 (7.4–9.3)
CBC Hct ^6^ [%]	21 (19–21)
ABG Hct [%]	20 (19–21)
SaO_2_ ^7^ [%]	98 (97–99)
SpO_2_ ^8^ [%]	97 (94–99)
PI ^9^	3.2 (2.1–5.4)

^1^ Interquartile range; ^2^ complete blood count; ^3^ hemoglobin; ^4^ arterial blood gas; ^5^ non-invasive hemoglobin determination; ^6^ hematocrit; ^7^ arterial oxygen saturation; ^8^ peripheral oxygen saturation; ^9^ perfusion index.

## Data Availability

The data presented in this study are available on request from the corresponding author.

## References

[1] Vincent J.L., Baron J.F., Reinhart K., Gattinoni L., Thijs L., Webb A., Meier-Hellmann A., Nollet G., Peres-Bota D., ABC (Anemia and Blood Transfusion in Critical Care) Investigators (2002). Anemia and blood transfusion in critically ill patients. JAMA.

[2] Corwin H.L., Gettinger A., Pearl R.G., Fink M.P., Levy M.M., Abraham E., MacIntyre N.R., Shabot M.M., Duh M.-S., Shapiro M.J. (2004). The CRIT Study: Anemia and blood transfusion in the critically ill—current clinical practice in the United States. Crit. Care Med..

[3] Thomas J., Jensen L., Nahirniak S., Gibney R.T. (2010). Anemia and blood transfusion practices in the critically ill: A prospective cohort review. Heart Lung.

[4] Koch C.G., Li L., Sun Z., Hixson E.D., Tang A., Phillips S.C., Blackstone E.H., Henderson J.M. (2013). Hospital-acquired anemia: Prevalence, outcomes, and healthcare implications. J. Hosp. Med..

[5] Holland J., Peralta R.M., Moss R.L., Feane K., Uprichard J. (2020). A single-centre review of iatrogenic anaemia in adult intensive care. Transfus. Med..

[6] Hayden S.J., Albert T.J., Watkins T.R., Swenson E.R. (2012). Anemia in critical illness: Insights into etiology, consequences, and management. Am. J. Respir. Crit. Care Med..

[7] Czempik P.F., Wilczek D., Herzyk J., Krzych Ł.J. (2022). Hospital-Acquired Anemia in Patients Hospitalized in the Intensive Care Unit: A Retrospective Cohort Study. J. Clin. Med..

[8] Meybohm P., Richards T., Isbister J., Hofmann A., Shander A., Goodnough L.T., Muñoz M., Gombotz H., Weber C.F., Choorapoikayil S. (2017). Patient Blood Management Bundles to Facilitate Implementation. Transfus. Med. Rev..

[9] Gayat E., Bodin A., Sportiello C., Boisson M., Dreyfus J.-F., Mathieu E., Fischler M. (2011). Performance evaluation of a noninvasive hemoglobin monitoring device. Ann. Emerg. Med..

[10] Berkow L., Rotolo S., Mirski E. (2011). Continuous noninvasive hemoglobin monitoring during complex spine surgery. Anesth. Analg..

[11] Applegate R.L., Barr S.J., Collier C.E., Rook J.L., Mangus D.B., Allard M.W. (2012). Evaluation of pulse cooximetry in patients undergoing abdominal or pelvic surgery. J. Am. Soc. Anesthesiol..

[12] Huang P., Shih B., Tsai Y.-F., Chung P., Liu F., Yu H., Lee W., Chang C., Lin C. (2016). Accuracy and Trending of Continuous Noninvasive Hemoglobin Monitoring in Patients Undergoing Liver Transplantation. Transplant. Proc..

[13] Joseph B., Pandit V., Aziz H., Kulvatunyou N., Zangbar B., Tang A., Keeffe T.O., Jehangir Q., Snyder K., Rhee P. (2015). Transforming hemoglobin measurement in trauma patients: Noninvasive spot check hemoglobin. J. Am. Coll. Surg..

[14] Awada W.N., Mohmoued M.F., Radwan T.M., Hussien G.Z., Elkady H.W. (2015). Continuous and noninvasive hemoglobin monitoring reduces red blood cell transfusion during neurosurgery: A prospective cohort study. Int. J. Clin. Monit. Comput..

[15] Riess M.L., Pagel P.S. (2016). Noninvasively Measured Hemoglobin Concentration Reflects Arterial Hemoglobin Concentration Before but Not After Cardiopulmonary Bypass in Patients Undergoing Coronary Artery or Valve Surgery. J. Cardiothorac. Vasc. Anesth..

[16] Welker E., Novak J., Jelsma L., Koehler T., Davis A., DeCou J., Durkin E. (2018). Continuous hemoglobin monitoring in pediatric trauma patients with solid organ injury. J. Pediatr. Surg..

[17] Frasca D., Dahyot-Fizelier C., Catherine K., Levrat Q., Debaene B., Mimoz O. (2011). Accuracy of a continuous noninvasive hemoglobin monitor in intensive care unit patients. Crit. Care Med..

[18] Raikhel M. (2012). Accuracy of noninvasive and invasive point-of-care total blood hemoglobin measurement in an outpatient setting. Postgrad. Med..

[19] DeBarros M., Shawhan R., Bingham J., Sokol K., Izenberg S., Martin M. (2015). Assessing serum hemoglobin levels without venipuncture: Accuracy and reliability of Pronto-7 noninvasive spot-check device. Am. J. Surg..

[20] Czempik P.F., Spień A., Oleksa M., Wiśniewski D., Krzych L.J. (2022). Inpatient recipients of packed red blood cells in a university medical center in Poland in 2018–2019. J. Transfus. Med..

[21] Gomaa D., Rodriquez D., Petro M., Blakeman T.C., Branson R.D. (2017). Impact of Oxygenation Status on the Noninvasive Measurement of Hemoglobin. Mil. Med..

[22] Lee S.W. (2022). Regression analysis for continuous independent variables in medical research: Statistical standard and guideline of Life Cycle Committee. Life Cycle.

[23] Hebert P.C., Wells G., Blajchman M.A., Marshall J., Martin C., Pagliarello G., Tweeddale M., Schweitzer I., Yetisir E., Transfusion Requirements in Critical Care Investigators for the Canadian Critical Care Trials Group (1999). A Multicenter, Randomized, Controlled Clinical Trial of Transfusion Requirements in Critical Care. N. Engl. J. Med..

[24] Zhang W., Zheng Y., Yu K., Gu J. (2021). Liberal transfusion versus restrictive transfusion and outcomes in critically ill adults: A meta-analysis. Transfus. Med. Hemother..

[25] Shabaninejad H., Ghadimi N., Sayehmiri K., Hosseinifard H., Azarfarin R., Gorji H.A. (2019). Comparison of invasive and noninvasive blood hemoglobin measurement in the operating room: A systematic review and meta-analysis. J. Anesth..

